# Bundles of Spider Silk, Braided into Sutures, Resist Basic Cyclic Tests: Potential Use for Flexor Tendon Repair

**DOI:** 10.1371/journal.pone.0061100

**Published:** 2013-04-17

**Authors:** Kathleen Hennecke, Joern Redeker, Joern W. Kuhbier, Sarah Strauss, Christina Allmeling, Cornelia Kasper, Kerstin Reimers, Peter M. Vogt

**Affiliations:** 1 Department of Plastic, Hand, and Reconstructive Surgery, Medical School Hannover, Hannover, Germany; 2 Institute of Applied Microbiology, University of Natural Resources and Life Sciences, Vienna, Austria; University of Notre Dame, United States of America

## Abstract

Repair success for injuries to the flexor tendon in the hand is often limited by the in vivo behaviour of the suture used for repair. Common problems associated with the choice of suture material include increased risk of infection, foreign body reactions, and inappropriate mechanical responses, particularly decreases in mechanical properties over time. Improved suture materials are therefore needed. As high-performance materials with excellent tensile strength, spider silk fibres are an extremely promising candidate for use in surgical sutures. However, the mechanical behaviour of sutures comprised of individual silk fibres braided together has not been thoroughly investigated. In the present study, we characterise the maximum tensile strength, stress, strain, elastic modulus, and fatigue response of silk sutures produced using different braiding methods to investigate the influence of braiding on the tensile properties of the sutures. The mechanical properties of conventional surgical sutures are also characterised to assess whether silk offers any advantages over conventional suture materials. The results demonstrate that braiding single spider silk fibres together produces strong sutures with excellent fatigue behaviour; the braided silk sutures exhibited tensile strengths comparable to those of conventional sutures and no loss of strength over 1000 fatigue cycles. In addition, the braiding technique had a significant influence on the tensile properties of the braided silk sutures. These results suggest that braided spider silk could be suitable for use as sutures in flexor tendon repair, providing similar tensile behaviour and improved fatigue properties compared with conventional suture materials.

## Introduction

Hand injuries are one of the most frequent forms of trauma. More than one third of all professional accidents result in hand injuries [1], [2]. The injuring of flexor tendons is particularly problematic and often results in such functional disorders as necrosis and adhesions caused by connective tissue and capillary revascularisation.

After the application of optimal surgical techniques and early-functional training with Kleinert splints [2], [3], the use of surgical sutures often causes problems. Sutures might tear, decrease in tensile strength, cause foreign body reactions, and encourage infections [2], [4].

Tendon tissue is characterised by high strength, elasticity, and plasticity; low strain (5–10%); and a failure stress of approximately 60–120 *MPa* [5], [6]. Force-strain curves show a characteristic, curve progression that is first almost exponential and then becomes nearly linear [6], [7].

Although a wide range of surgical sutures exist for all types of use, they all share certain characteristics. All surgical sutures must have high tensile strength and high knot security. Not only knot-slippage but also infections depend on the surface of the material used. Sterilisation and biocompatibility are especially important for use in human bodies [8].

Beyond these common features, plurality in application requires plurality in the choice and nature of suture materials. The readaptation of flexor tendon injuries demands a suture with high tensile strength but also low risk of tearing of the tendon [2] and dehiscence of the tendon stumps under functional training. Perfusion should not be disturbed by tendon strangulation, and irritation and adhesion should be avoided [2], [4].

These problems result in continuing search for new suture materials.

Spider silk is an outstanding biomaterial. The dragline silk of the golden orb web spider *Nephila clavipes* shows high tensile strength and elasticity combined with low density [9], [10]. Its biocompatibility has been recently described [10], [11], although it remains a matter of dispute [12]. Hakimi et al. reported the inhibition of cell growth on spider egg sacs due to toxic components, whereas different studies have shown that cell proliferation and adhesion are supported when the silk is harvested directly from the spider [13], [14], [15].

Because spider silk has been of interest for some time, its mechanical and thermal properties have been investigated in detail, as has its protein structure. Spiders can produce up to seven different types of silk, each with a special use and different protein composition. Of these types, dragline silk is the strongest [9], [16], [17], making it the focus of a large volume of research. Depending on the reference, *Nephila clavipes* silk resists a stress of 700–1700 *MPa* [9]. Its elasticity varies between 17–39% [9], [10], [18], and its elastic modulus reaches values up to 13 *GPa* [9], both depending on the relative humidity (see below).

The stress-strain curves of spider silk show a characteristic progression, similar to that of tendon, with a low-strain modulus and a high-strain modulus. Interestingly, spider silk also seems to have a “memory”-shape effect, allowing it to recover its initial form after deformation [19], similar to tendon, which should maintain or recover its form after exposure.

Spider silk also exhibits supercontraction. In reaction to its environmental conditions, spider silk contracts after water uptake to up to 50% of its initial length [20], [21]. The water molecules interact with spider silk proteins in local regions within the silk, with hydrogen bonding playing an important role. This interaction leads to a permanent change in the molecular composition of the silk, which results in changes in the silk’s biomechanical behaviour, such as induced stress and rubber-like increased elasticity [20], [21], [22].

Although the failure stress and strain of spider silk is higher than that of tendon, both materials show similar characteristic curve progressions under stress. Both materials tend to keep or recover their form after stress, and both function under wet conditions: spider silk due to the effect of supercontraction and tendon due to its natural environment. These features make these materials an interesting and promising match.

Main aims of the following study.

As single spider silk fibres are a high-performance material with excellent tensile strength, we opted to examine their biomechanical characteristics with regard to future use as surgical sutures. A preliminary study of 30 single spider silk fibres has previously been made [23]. In this context, we asked whether the mechanical properties of single fibres are preserved after being processed into sutures containing a larger quantity of fibres (up to 12 times of the original number of 30). Is the mechanical behaviour of braided dragline spider silk sutures influenced by the chosen type of braiding? All sutures were tested with regard to failure load, stress, strain, and elastic modulus. Five commercially available surgical sutures of different gauge numbers were tested under identical conditions to obtain data for the comparison of mechanical behaviour: Prolene® (polypropylene), PDS II® (polydioxanone), Ethilon® (polyamide 6), Maxon™ (polyglyconate), and Vicryl® (polyglactin).

Cyclic tests to imitate continuous use were conducted in the second part of this study. Spider silk and conventional suture material were tested for significant differences in resistance to cyclic use regarding failure load and an increase in strain.

## Materials and Methods

### Animal Care

Spiders were kept at a local animal care unit. The animals were fed regularly, approximately three times a week, with crickets and watered daily with tap water. To keep the room climate ideal, the atmospheric humidity was set to approximately 70%. Special daylight lamps were used during the winter months. Silk was harvested from female adult Nephila spiders ranging in age from 6 to 15 months.

### Silk Rearing and Storage

Spider silk was collected by the method recently described in Kuhbier et al. [15], (figure S1). The silking speed was set to 4 *cm/sec*. To reduce the influence of material differences, only 20 single fibres were taken from each spider. These fibres were combined into bundles of 60 to 120 single fibres. Bundles for subsequent braiding and the resulting braided sutures were strainlessly wound on large polypropylene tubes and stored under the same environmental conditions as the spiders: namely, room temperature and a constant air humidity. The bundles of silk were stored for up to six months before being processed into sutures, and the sutures were stored for up to three months before testing.

### Braiding of the Silk Fibres

Spider silk sutures were made with the use of a miniature rope machine with a maximum capacity to intertwine seven silk strands of fibres, as recently described by Kuhbier et al. [23], (figure S2). For all studies, the angle at which the strands were put together was chosen as between 25° and 35° from horizontal. Table 1 shows the definition of the nomenclature of sutures.

**Table 1:** Definition of the nomenclature of sutures.

Fibre	A single filament of dragline spider silk.
Bundle	A bunch of native spider silk fibres.
Strand	A bundle of spider silk fibres processed in a braided suture.
Suture	A braided combination of strands.

To compare different types of braiding, sutures were prepared from either three strands and varying numbers of single fibres (3×60–3×120 single fibres) or varying numbers of strands (3×60 to 6×60 single fibres). The resulting sutures were stored until biomechanical testing.

### Scanning Electron Microscopy and Diameter of the Braided Suture

The tightness and thickness of the ropes as well as single fibre diameters were averaged over approximately 15 chosen samples using scanning electron microscopy (SEM).

Samples of 1 *cm* braided sutures or bundles of single threads were coated with gold using an argon sputterer (SEM Coating System, Polaron, East Grinstead, United Kingdom). The specimens were fixed in a vacuum and viewed via SEM (SEM500, Philips, Hamburg, Germany) at magnifications ranging from 20× to 400× at a voltage of 10 *kV*, visualised by scanning with 1000 horizontal lines per image with a 32 *m*s scanning time per image, resulting in a resolution of 1333×1000 pixels. Single fibres were measured using software developed by Gebert & Preiss. As the drill of the sutures tended to untwist immediately after the manufacturing process, leading to unwanted spaces between the strands, sutures were glued on both sides of the specimen devices. Nevertheless, these spaces could not be avoided completely, rendering it impossible to determine the diameter of the ropes using SEM; instead, a theoretic value based on over 100 single fibre diameters was used.

Spider silk is a biomaterial. As this biomaterial is influenced by the food, season, and age of the spider, the single fibre diameters of silk may vary. To minimise the effect of this variation, silk from at least nine different spiders was used for the braiding of each suture. To obtain more accurate values of the diameters of these sutures, over 100 single fibre diameters were measured using SEM. Accurate knowledge of the suture diameters is important for defining a cross-section area of the suture when calculating the stress in biomechanical tests. Based on the single fibre diameters, a theoretical diameter was calculated using the equation for the cross-section area A of a circle with radius r (figure S3).

The calculated radius was used to define an approximated cross-section area for braided sutures of spider silk. The diameters were calculated for dry sutures.

### Size Measurement of Wet and Dry Braided Spider Silk Sutures

The effect of supercontraction for spider silk has been frequently described [24], [25]. Wet silk contracts immediately after water uptake [20], [21]. As this is clearly an important aspect for the use of spider silk in the human body, all braided sutures were measured under wet conditions. An initial suture length of 30 *cm* was defined under dry conditions. Specimens were moistened with 0.9% NaCl to mimic physiological conditions. After moistening, the resulting lengths were measured.

Because no supercontraction-like phenomenon has been described for commercially available sutures, these materials were tested dry.

### Biomechanical Tests

All sutures were tested with an Instron® testing system, model 5565A. Samples were clamped with pneumatic cord and yarn grips. In this way, samples breaking at the grip of the clamp was minimised. Samples that still broke near the grip were excluded from analysis.

For each tested suture, the failure load, stress, strain, and elastic modulus were measured and calculated:

Load *F* in Newton 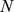:
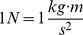
(1)


Failure stress 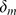 in *MPa*:
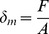
(2)


Failure strain 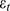, depending on initial length *L_0_* and elongation *ΔL*, in *mm/mm*:
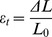
(3)


Elastic modulus *E* in *MPa*, Hooke’s Law:
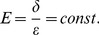
(4)


The elastic modulus is the slope of the stress-strain curve in the elastic region.

Tests started after reaching a low preload of 0.05 *N*. The testing speed was 30 *mm/min* until break, with a load cell of 5 *kN*. Tests were executed at room temperature.

Prolene® 6-0 and spider silk 3×120 single fibres were chosen for cyclic testing. The spider silk was held wet in the cyclic testing, which was guaranteed by a medical infusion system and a special construction. Both materials were subjected to 1000 cycles between zero and half of their mean maximum load. This number of cycles was chosen based on the assumption of post-surgical training of 5× approximately 30 cycles of practice each week over a period of 6–7 weeks. Immediately after the last cycle, the suture was stretched until break. To compare the influence of cyclic testing on the biomechanical qualities of these two materials, the increase of elongation from the first to the last cycle was studied in addition to failure load.

### Statistical Analysis

The samples were checked for normal distributions using the Kolmogorov-Smirnov test and the D’Agostino and Pearson omnibus Test. For each value, the mean, standard deviation (SD), standard error of the mean (SEM) and variance were calculated.

The results of the effects of different types of braiding were checked by analysis of variance with the Bonferroni-Test and an unpaired t-test. The results before and after cyclic testing were also checked by an unpaired t-test.

Differences were stated as significant or highly significant if the probability p for type I error, i.e., incorrect rejection of the null hypothesis, was equal to or less than the chosen levels of significance: 0.05(*), 0.01(**), or 0.001(***).

## Results

### Braided Sutures of Spider Silk

**Figure 1:**
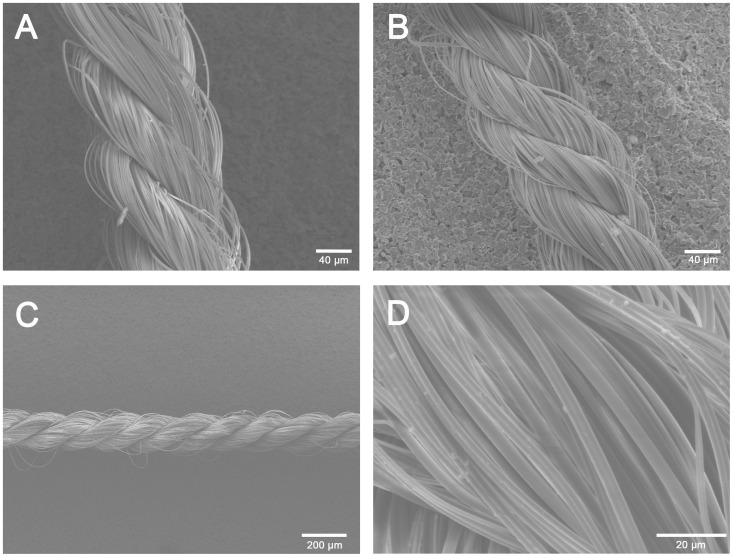
SEM of single spider silk fibres and braided spider silk sutures. A shows a braided suture of 4×60 single spider silk fibres. Fibres are clearly visible in their corresponding strands. Strands are at an angle of approximately 30° from horizontal. B shows a braided suture of 3×120 single silk fibres. C depicts a large section of harmonic braiding. D presents a typical example of the SEM images used for diameter measurements.

Spider silk could be used to create sutures with different strand compositions. These silk sutures were prepared as polyfil threads consisting of up to 6 strands braided by a specifically designed braiding apparatus [23], (figure S2). The resulting threads show an extraordinarily regular pattern, as shown by the SEM images in figure 1 A–C. Figure 1 A shows a braided suture of 4×60 single spider silk fibres. Single fibres are clearly visible in regularly intertwined bundles. The strands are at an angle of approximately 30° from horizontal. Figure 1 B shows a braided suture of 3×120 single silk fibres. C shows a large section of harmonic braiding.

To calculate the tensile strength of the fibres for comparison with that of commercially available suture material, the diameters of the braided sutures had to be determined. Because spider silk is a highly variable biomaterial, the diameters of the laid sutures were determined by measuring single fibre diameters from SEM images. In all, 105 single spider silk fibres were measured using SEM, with a mean diameter of 1.994 *µm* ±0.37 (SD) (figure 1 D). The diameters for the braided sutures are given in table 2.

**Table 2:** Tested braided spider silk and commercially available sutures with mean diameters in mm.

Single fibres	Gauge	Spider silk	Prolene®	PDS® II	Ethilon®	Maxon™	Vicryl®
**3×60**	**6-0**	0.084	0.085				
**4×60/3×80**	**6-0**	0.097	–				
**5×60/3×100**	**5-0**	0.109	0.125			–	
**6×60/3×120**	**5-0**	0.119	–				
**–**	**4-0**	–	0.175		–		
**–**	**3-0**	–	0.225	–		0.225	-

The diameters of the braided spider silk sutures correspond to commercial suture materials. As a result, spider silk threads consisting of 180 single fibres corresponded to a mean diameter of 6-0 gauge suture material (0.07–0.099 *mm*), while the use of 240 fibres resulted corresponded to the upper range of 6-0 gauge. Additionally, 300 fibres and 360 fibres both achieved 5-0 gauge (0.1–0.149 *mm*), with 300 fibres residing in the lower range and 360 in the middle of the range. The 4-0 gauge includes sutures between 0.15–0.199 *mm*, whereas the 3-0 gauge includes sutures between 0.2–0.249 *mm*.

All diameters of the surgical sutures were mean diameters, chosen from the metric interval of each suture (table 2).

### Supercontraction of Wet Spider Silk Sutures

**Figure 2:**
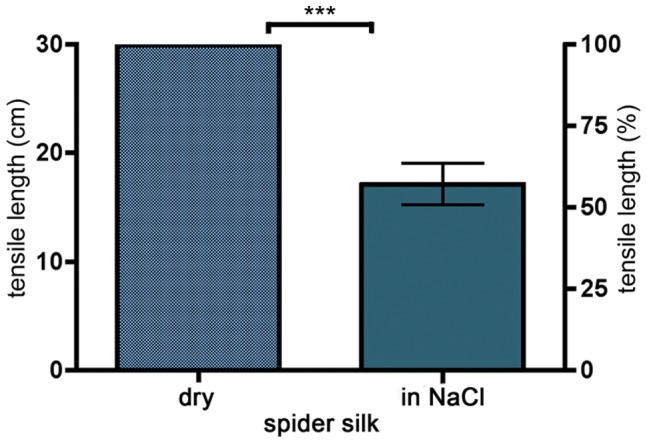
Supercontraction of braided sutures. Braided sutures were measured under wet conditions after being moistened with 0.9% NaCl. The resulting supercontraction (reduction of length) of the braided sutures is clearly visible. The results are highly significant, with p<0.0001.

Braided spider silk sutures were measured under wet conditions, moistened with NaCl 0.9% to mimic physiological conditions. The resulting supercontraction of braided sutures is shown in figure 2. Wet braided spider silk sustains a reduction to 57.1% ±6.4 (SD) of its initial length. All subsequent experiments were performed with moistened spider silk for standardised conditions.

### Tensile Testing

**Figure 3:**
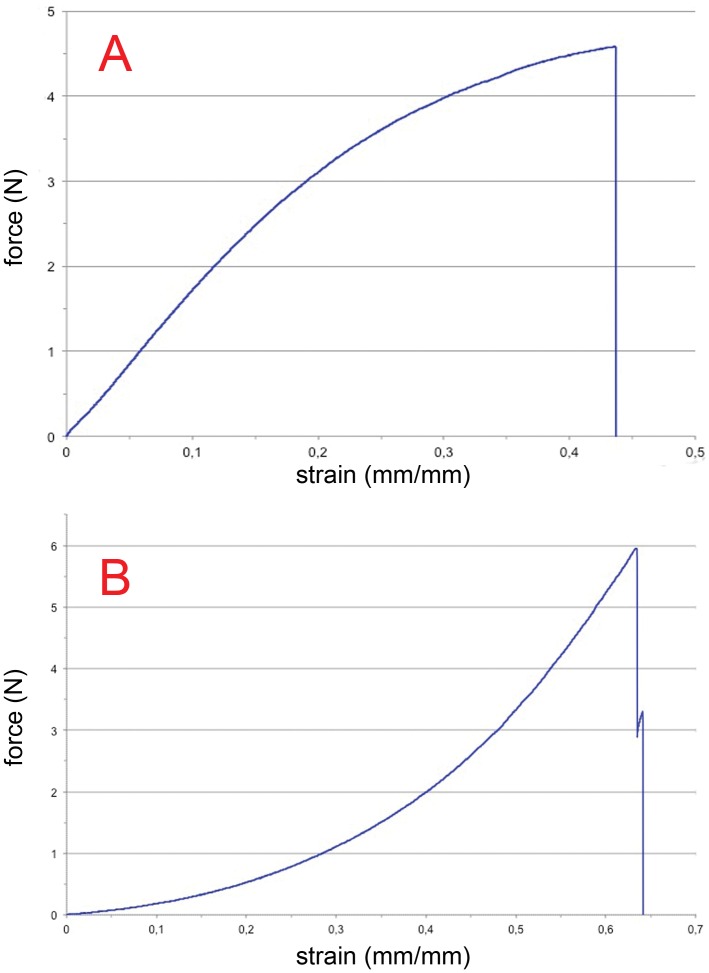
Tensile tests of Prolene® 6-0 and 3×120 spider silk. A shows the results of the tensile tests of a median specimen of Prolene® 6-0 with its corresponding force-strain curve. The curve starts with an almost linear low strain part and flattens in the second half. B shows the results of a median specimen of 3×120 braided spider silk with its corresponding force-strain curve. The curve is initially almost exponential, becoming nearly linear progression in the second half. Note that after reaching the failure load, not all fibres break simultaneously.

The failure force in *N* and the failure stress in *MPa* were measured. The elastic modulus was determined as the tangent of the curves. The results for the tensile testing of Prolene® 6-0 are shown in figure 3 A.

The force-strain curves of braided spider silk display a typical progression, examples of which are shown for 3×120 fibres in figure 3 B. In the beginning, the wet sutures can be stretched easily, as the curves rise moderately. After reaching about half-maximal extension, increasing force is necessary to continue stretching the suture with linear curve progression. This point is also where the elastic modulus for braided sutures of spider silk is expressed by the ratio of stress to strain as the slope of the linear curve. Interestingly, after reaching the maximum load, not all fibres break at the same time, leading to the discontinuous rupture curves in figure 3 B.

Surgical sutures were measured under identical testing conditions, except for being in the dry state, to determine their basic mechanical properties. A detailed discussion of the biomechanical properties of commercially available sutures was not intended. The force-strain curve of Prolene® 6-0 is shown in figure 3 A. The curve starts with a constant grading, so the elastic modulus is, in this case, measured for the first half of the testing.

### The Braiding Modus Influences the Mechanical Properties of the Spider Silk Sutures

**Figure 4:**
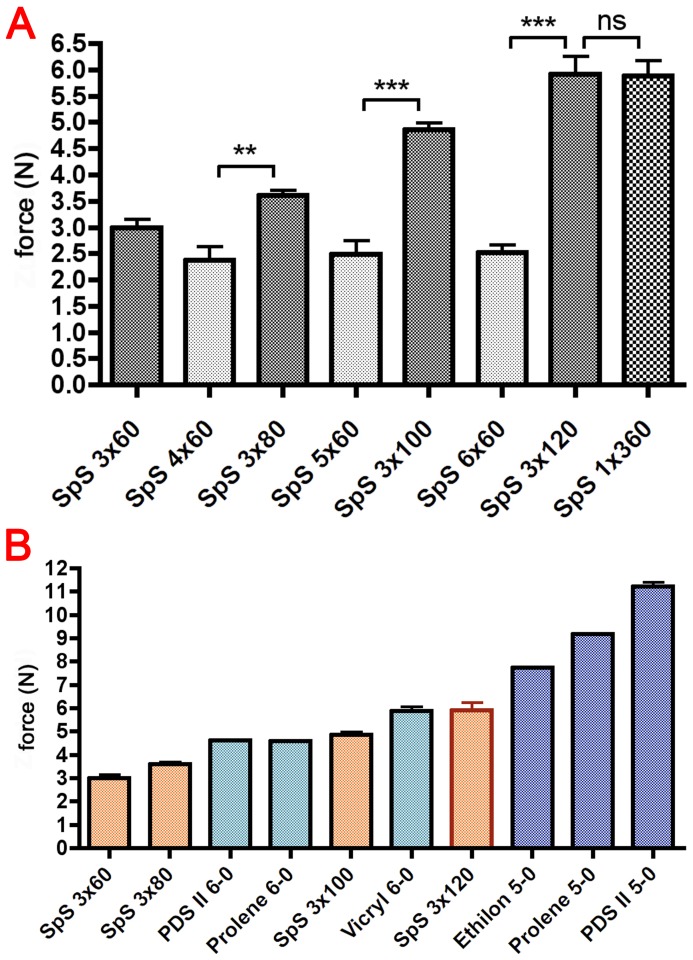
Comparison of different types of braiding of spider silk and surgical sutures. A compares two types of braiding of spider silk, the braiding of different numbers of silk bundles of 60 single fibres (3×60–6×60) and three bundles of silk with an increasing number of spider silk fibres (3×60–3×120). Asterisks indicate the significance level: p<0.01 (**) and p<0.001 (***). B compares the results for spider silk with those for surgical sutures to find a pair with almost identical failure load. It was found that 3×120 spider silk should be able to replace all tested sutures of gauge number 6-0.

The braided spider silk sutures were organised in two different ways to achieve standardised fibre counts. While one type of sutures had a fixed number of silk bundles and varying numbers of fibres per bundle (3×60, 3×80, 3×100, 3×120), the second type consisted of different numbers of bundles (3–6) with 60 fibres (table 2). As a control, 360 single fibres were used additionally. The results of the tensile testing of all spider silk sutures are summarised in tables 3 and 4 and visualised in figure 4 A. Interestingly, the braiding of different numbers of silk bundles of 60 single fibres (3×60–6×60) did not increase the maximum force and stress despite the increase in the number of fibres per thread. On the other hand, three bundles of silk with an increasing number of spider silk fibres (3×60–3×120) presented a constant increase of failure load. The production of 6×60 single spider silk fibres was stopped after the first results of testing due to poor mechanical performance. In contrast, the failure loads of the 3×120 braided single fibres and the 360 unbraided native spider silk fibres did not differ.

**Table 3:** Tensile test results for spider silk sutures of 3×60–6×60 single fibres.

	3×60	4×60	5×60	6×60
n	12	9	10	4
**failure load (N)**	2.99	2.37	2.49	2.52
**SD**	0.56	0.79	0.82	0.31
**failure strain (mm/mm)**	0.55	0.53	0.48	0.47
**SD**	0.07	0.16	0.08	0.05
**failure stress (MPa)**	581.0	316.5	266.8	226.6
**SD**	145	107.9	87.3	27.6
**modulus**	2.01	1.30	1.04	0.62
**SD**	0.75	0.90	0.43	0.13

**Table 4:** Tensile test results of spider silk sutures of 3×60–3×120 single fibres and 1×360 native spider silk fibres.

	3×60	3×80	3×100	3×120	1×360
n	12	12	12	12	12
**failure load (N)**	2.99	3.61	4.86	5.92	5.89
**SD**	0.56	0.34	0.44	1.16	1.01
**failure strain (mm/mm)**	0.55	0.56	0.60	0.65	0.68
**SD**	0.07	0.05	0.05	0.05	0.08
**failure stress** **(MPa)**	581.0	464.5	495.1	532.2	528.3
**SD**	145	61.4	67.1	104.2	91.8
**modulus**	2.01	1.74	1.84	1.68	1.69
**SD**	0.75	0.28	0.19	0.38	0.27

Braided spider silk was compared with surgical sutures to find a pair with almost identical failure loads. Figure 4 B shows that 3×120 spider silk should be able to replace all tested sutures of gauge number 6-0. This finding prompted us to compare spider silk and Prolene® 6-0 in the following cyclic tests.

### Cyclic Testing of 3×120 Spider Silk and Prolene® 6-0

**Figure 5:**
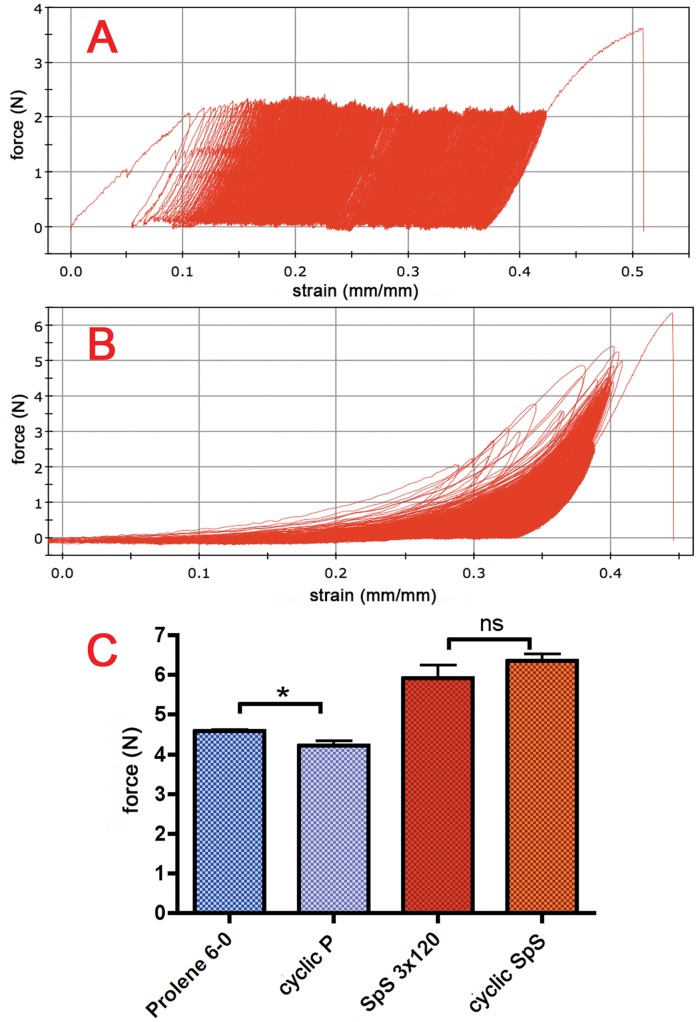
Results for cyclic tests of Prolene® 6-0 and 3×120 spider silk. A shows the cyclic testing results for Prolene® 6-0. The suture was subjected to over 1000 cycles between 0 *N* and 2.3 *N*, which was half of the mean failure load. After these 1000 cycles, Prolene® was stretched up to breaking point. The failure load after cyclic testing was compared with the previous single tensile testing (C). The results of biomechanical tests of 3×120 spider silk are shown in B. The first 100 to 300 cycles of testing did not remain under the limit of half of the failure load of 2.9 *N*. No difference in failure load before and after cyclic use was noticed.

Considering that suture materials used for repair of musculoskeletal tissues, e.g., tendons, must resist continued mechanical load, as long terms of immobilisation are avoided in modern therapeutic schemes [3], [26], our next step was to test whether our braided spider silk suture material is able to withstand repeated expansion. To this end, we performed a cyclic testing protocol of 1000 elongation steps up to the half-maximal load as determined before. Figure 5 A shows the results of the cyclic testing of Prolene® 6-0 with 1000 cycles between 0 *N* and 2.3 *N*, which was half of the mean maximum load. The polyethylene was stretched constantly from one cycle to the next and did not revert to zero strain, even if no force was applied.

In contrast, the spider silk strain returned to zero after relaxation (figure 5 B). During the testing, this relaxation decreased. The first 100 to 300 cycles of testing did not remain below the limit of half of the mean maximum load of 2.9 *N*, which could not be avoided.

After finishing 1000 cycles, both materials were stretched to the breaking point. The maximum load after cyclic testing was compared with previous single tensile testing in table 5 and 6 and visualised in figure 5 C. Additionally, we determined the increase in the strain for the half-maximal load because in case of spider silk, the strain and relaxation after biomechanical testing could not be determined exactly for the point when no force was applied due to the overlap of the measurements.

**Table 5:** Maximum load before and after cyclic testing for Prolene® 6-0 with increasing strain between the first and last cycle.

	Single testing	Cyclic testing
n	10	10
**failure load (N)**	4.58	4.22
**SD**	0.14	0.37
**increase of strain (mm/mm)**	–	0.24
**SD**	–	0.062

**Table 6:** Maximum load before and after cyclic testing for spider silk sutures comprised of 3×120 single fibres with an increase in strain between the first and last cycle.

	Single testing	Cyclic testing
n	12	10
**failure load (N)**	5.92	6.35
**SD**	1.16	0.52
**increase of strain (mm/mm)**	–	0.072
**SD**	–	0.015

The failure load of Prolene® decreased significantly after cyclic testing. Spider silk, on the other hand, did not seem to be influenced by cyclic testing. Compared to single testing, the failure load after cyclic testing was essentially the same. Regarding the increase in strain, Prolene® showed an increase of 24% ±1.9 under cyclic testing, whereas spider silk presented an increase of only 7.2% ±0.48.

## Discussion

We developed a braiding method that led to strong spider silk sutures with a constant increase in failure load with fibre number per bundle. These results were confirmed by comparison with the failure load of unbraided bundles of native spider silk. An important point in the conceptual design of our experiments is that spider silk is a natural product that is influenced by its environmental conditions, e.g., prey composition. These material differences were addressed by employing optimised farming conditions and low number of single fibres taken from one spider to reduce differences in the thicknesses of single fibres and braided sutures [27], [28], [29], [30].

The failure load of the braided sutures was lower than expected based on single fibre maximum load data in the literature. For instance, Vehoff et al. published data from Nephila dragline fibres with maximum loads between 30.0 and 36.5 *mN* [17]; based on their findings, a sum of 360 single fibres should theoretically be able to withstand a force of between 10.8 and 12.9 *N*. In our study, the corresponding values were between 3.7 of 5.89 *N*, as shown in table 4. This finding illustrates that these values are unfavourable for comparing the material under the chosen settings. The results of material stress based on diameter per cross-section area are much better suited for such comparisons.

Cunniff et al. reported that the maximum stress of single dragline fibres was 0.7 *GPa* [9], which was much nearer to our results for braided spider silk suture, with a maximum stress of 0.58 *GPa*.

Nevertheless, other authors have published different results, with stresses up to 1.0 *GPa* [31], [32], [33]. This discrepancy leads to the question of other influential parameters. Some variability doubtlessly originates in the spiders themselves, both in terms of species or even subspecies as well as whether the animals were farmed or caught in nature [33].

However, one of the most important parameters is the method of silk rearing. Forced silking, with or without anaesthesia, influences the production of silk [24], [32], [33]. Increased stress and the resulting heat during production might reduce spinning speed and silk quality [32], [18]. Thus, the spiders’ feelings of stress affect the single fibre diameters, which in turn affect the failure stress.

The results for micro sutures with 30 single fibres of spider silk in the same laboratory presented much higher stress values, with a value of 0.7±0.2 (SD) *MPa* for 2×15 strands and 0.8±0.3 (SD) *MPa* for 3×10 single strands [23]. In that study, the diameters of braided sutures could be scaled precisely on SEM, as the number of single fibres was reduced to 30. This method did not reveal a wide range of diameters, unlike the present work. For this reason, force in Newtons was chosen to find a corresponding pair of sutures for the following cyclic tests rather than stress, which depends on diameter. As 3×120 spider silk was able to replace all sutures of gauge 6-0, it was compared with Prolene® 6-0. Increasing friction and torsion within strands and the suture is another interesting aspect and should be clarified in future work on this subject.

Recombinant spider silk seems to avoid all of the problems with native spider silk. Its success is no longer confined to cell culturing [34], [35]. Wound dressing made from recombinant spider silk protein was recently described [36]. However, recent research seems to have concentrated on protein engineering. Recombinant spider silk is still not able to reproduce all of the biomechanical characteristics of the silk identically, although the similarity is improving [30]. Xia et al. described silk proteins produced by E. coli that could be used to produce silk fibres. The resulting fibres achieved similar results for stress and strain as native *N. clavipes* dragline silk. However, differences remained concerning the elastic modulus.

The elastic modulus is mainly influenced by material strain. As water uptake and supercontraction increase the strain, the values for the elastic modulus must decrease in inverse proportion. Very low values of the elastic modulus, i.e., between 3.2±0.4 *GPa* [21] and 1.5±0.5 *GPa* [17], for wet spider silk have already been described in the literature [17] and agree with the findings of this work (table 3 and 4). As spider silk sutures are intended for use in human bodies, the effect of supercontraction must be taken into consideration.

Biomechanical tests are important for the further use of spider silk sutures in tendon repair. Considering clinical demands, spider silk must be able to withstand cyclic testing. Vehoff et al. published data describing the relaxation of spider silk after initial elongation [17]. Single dragline fibres did not show a loss of maximum load after several cycles. The importance of water uptake in this context and the so-called viscoelasticity of silk has already been described by Gosline et al. [31]. In our study, two differences between spider silk and Prolene® must be noted in the cyclic test results. Polyethylene showed an increase of elongation of approximately 24% ±6.2, which was much higher than that for spider silk, which was 7.2% ±1.5. This finding shows that spider silk is less strongly affected by cyclic use than polyethylene. Emile et al. describe a shape ‘memory’ in spider dragline for torsion, which demonstrates impressively the ability of spider silk to retain its original shape. This effect might also explain the remarkably smaller increase of elongation of spider silk [19].

Even more interesting is the difference in the failure load before and after use. Prolene® presented a significant decrease in its failure load, whereas the failure load of spider silk did not seem to change at all. This consistency is ideal for tendon sutures, which must resist a constant strain.

Of course, a model over a testing period of less than 24 h cannot represent the physiology of the human body during the weeks and months of the healing process. However, these results suggest that spider silk is better suited for repairing tendons or any other body structure with biomechanical use.

Although the failure stress and strain of spider silk sutures remain higher than those of tendons, both materials show a characteristic and similar curve progression under stress. The initial low-strain modulus of single spider silk fibres is not present in spider silk sutures [17]. As previously demonstrated, this effect is observed in dry fibres, not supercontracted fibres [17], [21]. Thus, it is not surprising that supercontracted spider silk sutures begin with a high strain under low stress/force (figure 3). After an exponential-like progression, when the supercontracted fibres are most likely relaxed, the curve transitions into an almost linear progression, for which Hooke’s Law can be applied.

Prolene®, on the other hand, presents an oppositional curve progression, with low strain and elastic modulus at the beginning transitioning into high strain in the second part before reaching failure load. In contrast, the stress-strain curves of tendon show a similar progression to spider silk sutures [6], [37]. In conclusion, both spider silk and tendon keep or recover their form after stress, and both work under wet conditions. Under cyclic use, spider silk sutures also show a tendency to regain their initial length. The higher strain of spider silk might reduce such effects as tear through and strangulation of the tendon by the suture. These aspects make both materials a promising match for further study in this field.

Additionally, considering its favourable biocompatibility [11], [38], cell proliferation on spider silk might also help to support the healing of tendon injuries. Likewise, foreign body reactions can be reduced or even avoided by its low immunogenicity.

To date, spider silk has not been used commercially for biomedical or other applications, limited by spiders’ natures. Being predators, spiders cannot be kept in close quarters with their conspecifics. Farming, feeding, and material production still remain basic problems. The production of handmade sutures also currently inhibits the production of large numbers of sutures. These problems demonstrate a demand for the development of new production processes.

Although the protein structure of spider silk is known in detail, recombinant silk still does not represent all characteristics of native silk [30], thus making the use of native spider silk preferential. Spider silking facilities, such as those on Madagascar, are an appealing alternative [39].

### Conclusions

This work shows how spider silk can be braided into strong regular sutures. The chosen type of braiding has a tremendous influence on the biomechanics of the braided dragline silk suture. The extraordinary mechanical properties of single dragline silk fibres could not be preserved completely. Even under the optimal braiding method, such interactions as torsion and friction between the fibres were observed. Nevertheless, a continuous increase of the failure force was observed under the optimal method of braiding. Variations caused by material differences could be minimised.

Permanent use, imitated by cyclic testing, does not seem to influence spider silk, whereas the failure load of the commercial material Prolene® 6-0 decreased significantly after cyclic testing. This finding leads to the conclusion that spider silk has good potential as an alternative suture material. The use of spider silk offers the opportunity to avoid existing problems in tendon healing and other types of tissue reconstruction and suggests a readaptation with the use of an innovative suture material. Further studies should focus on the biocompatibility and biodegradation of the silk in vivo.

## Supporting Information

Figure S1 Diagram of silk harvesting. For silk harvesting, spiders were fixed on styropor cubes without the use of anaesthesia. The spiders were immobilised by a gauze cover fixed with small needles to a styropor cube. Silk was pulled out of the major ampullate gland, which is an adequate stimulus for production. Dragline fibres were reared on a device of 30 *cm* in diameter, and silking speed was set to 4 *cm/sec*.
(TIF)

Figure S2 Braiding of the silk fibres. Sutures of spider silk were constructed using a miniature rope machine with a maximum capacity to intertwine seven silk strands of fibres. Yarns were fixed on one side (A), while the free ends were taken together on the opposite side and also fixed (C). The device and bolt were fastened tightly with a setscrew. The rope was laid by the co-rotation of the free ends while the pooled ends turned in the opposite direction, directed by a guiding carriage (B). This procedure prevented strong eccentricity while providing the necessary stability. The velocity by which the ropes were laid and the use of the carriage determined the angle at which the strands were put together. For all studies, angles between 25° and 35° from horizontal were chosen. To compare different types of braiding, sutures were either prepared from either three strands and varying numbers of single fibres (3×60–3×120 single fibres) or varying numbers of strands on the other hand (3×60 to 6×60 single fibres). The resulting sutures were stored for following biomechanical tests.
(TIF)

Figure S3Theoretical diameter and cross-section area of the braided suture. Accurate knowledge of the diameters is important for defining a cross-section area of the suture when calculating the stress in biomechanical tests. Based on single fibre diameters, a theoretical diameter was calculated using the following equation. The cross-section area *A* of a circle with radius *r* is defined as, 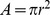 (1); The cross-section area *A_x_* as a sum of several single cross-section areas *A_1_* is defined as, 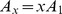 (2); After the insertion after formula 1, 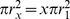 (3); Rearrangement yields 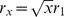 (4); The calculated radius is used to define an approximated cross-section area for braided sutures of spider silk. Figure S3 illustrates the calculation above.(TIF)
