# Supplementary figures and images for: Bundles of Spider Silk, Braided into Sutures, Resist Basic Cyclic Tests: Potential Use for Flexor Tendon Repair
Source: PLoS One. 2013 Apr 17;8(4):e61100. doi: 10.1371/journal.pone.0061100 (PMC3629086; doi:10.1371/journal.pone.0061100)

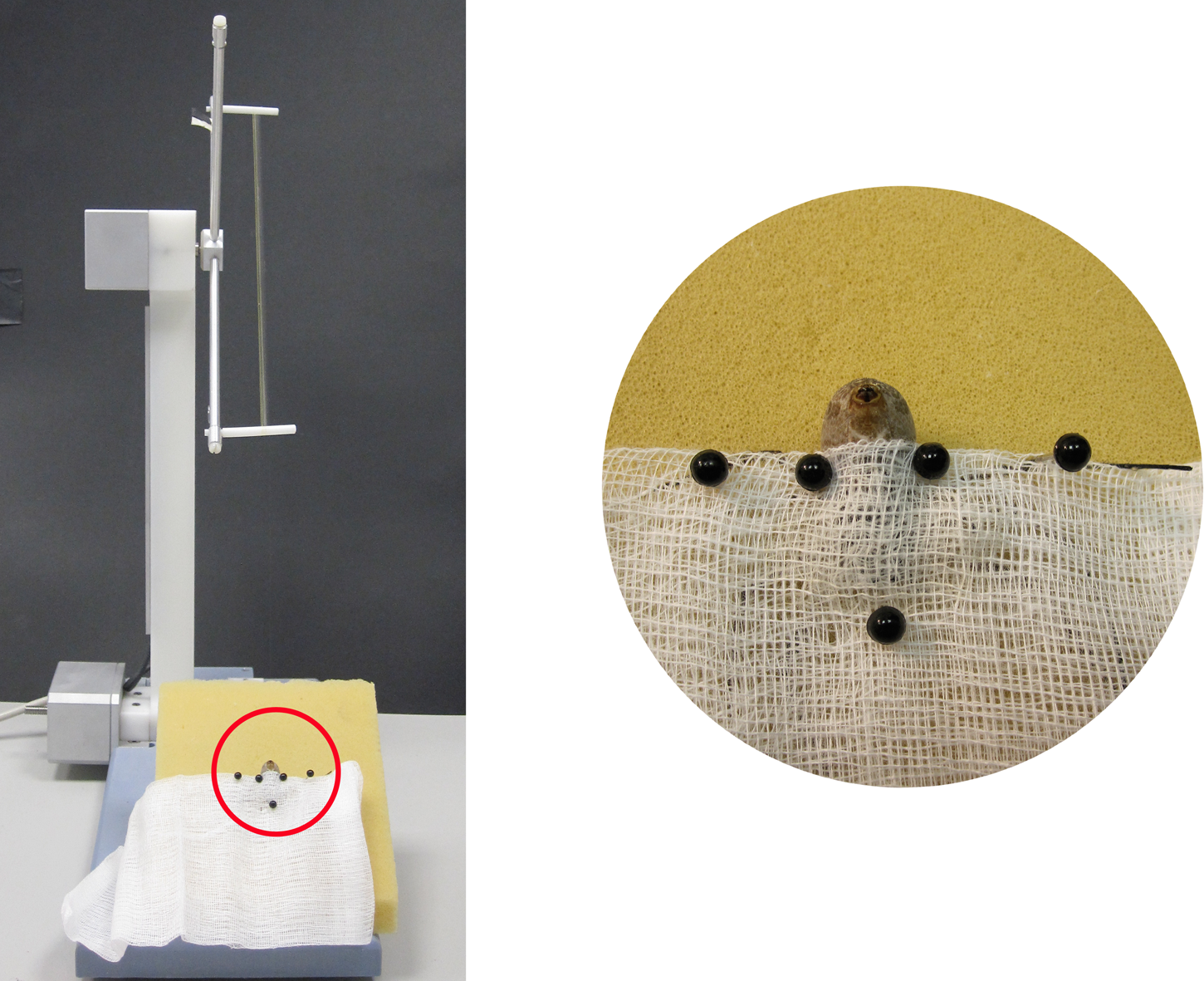

Supplement: Figure S1 — Diagram of silk harvesting. For silk harvesting, spiders were fixed on styropor cubes without the use of anaesthesia. The spiders were immobilised by a gauze cover fixed with small needles to a styropor cube. Silk was pulled out of the major ampullate gland, which is an adequate stimulus for production. Dragline fibres were reared on a device of 30 cm in diameter, and silking speed was set to 4 cm/sec. (TIF) [file pone.0061100.s001.tif]

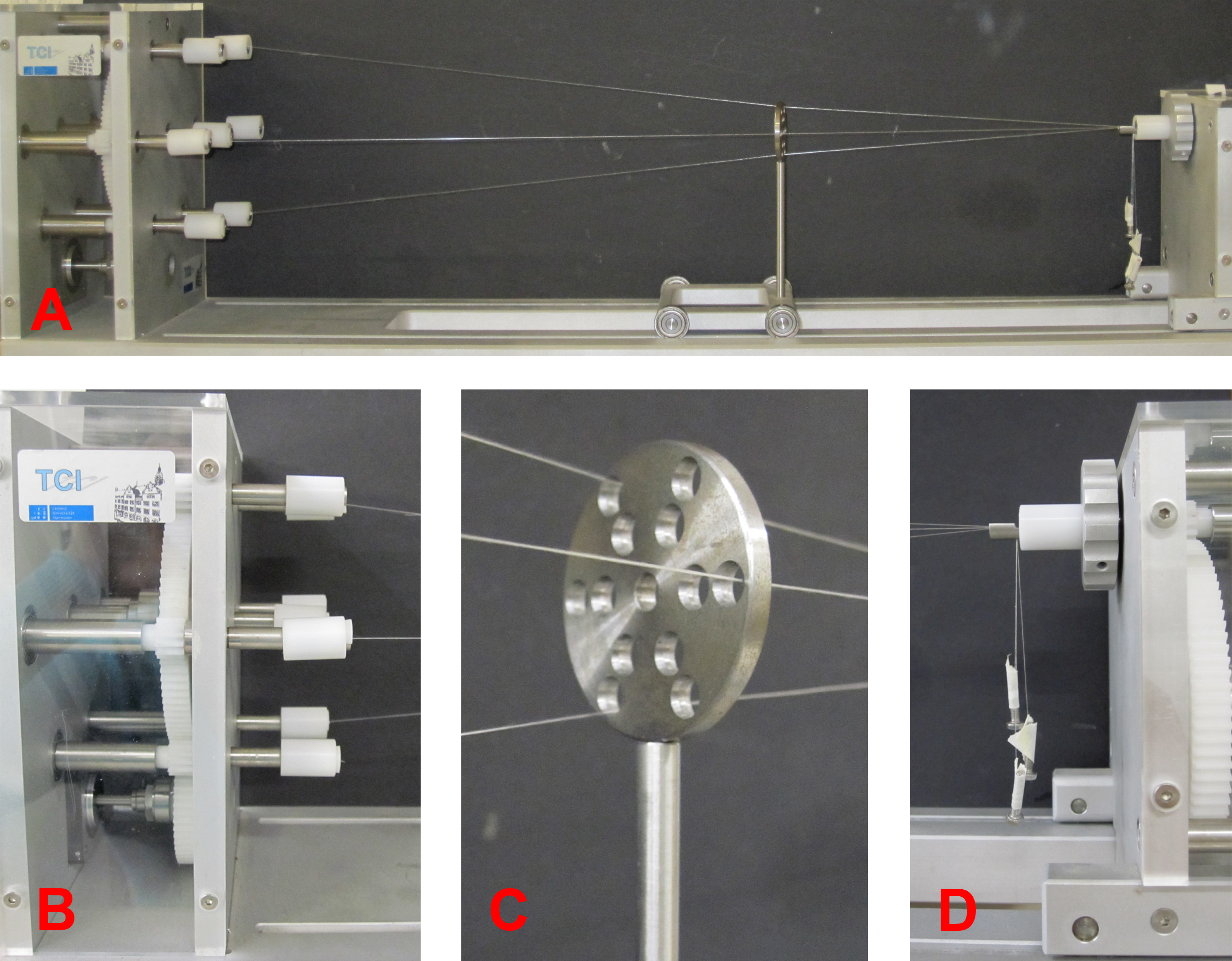

Supplement: Figure S2 — Braiding of the silk fibres. Sutures of spider silk were constructed using a miniature rope machine with a maximum capacity to intertwine seven silk strands of fibres. Yarns were fixed on one side (A), while the free ends were taken together on the opposite side and also fixed (C). The device and bolt were fastened tightly with a setscrew. The rope was laid by the co-rotation of the free ends while the pooled ends turned in the opposite direction, directed by a guiding carriage (B). This procedure prevented strong eccentricity while providing the necessary stability. The velocity by which the ropes were laid and the use of the carriage determined the angle at which the strands were put together. For all studies, angles between 25° and 35° from horizontal were chosen. To compare different types of braiding, sutures were either prepared from either three strands and varying numbers of single fibres (3×60–3×120 single fibres) or varying numbers of strands on the other hand (3×60 to 6×60 single fibres). The resulting sutures were stored for following biomechanical tests. (TIF) [file pone.0061100.s002.tif]

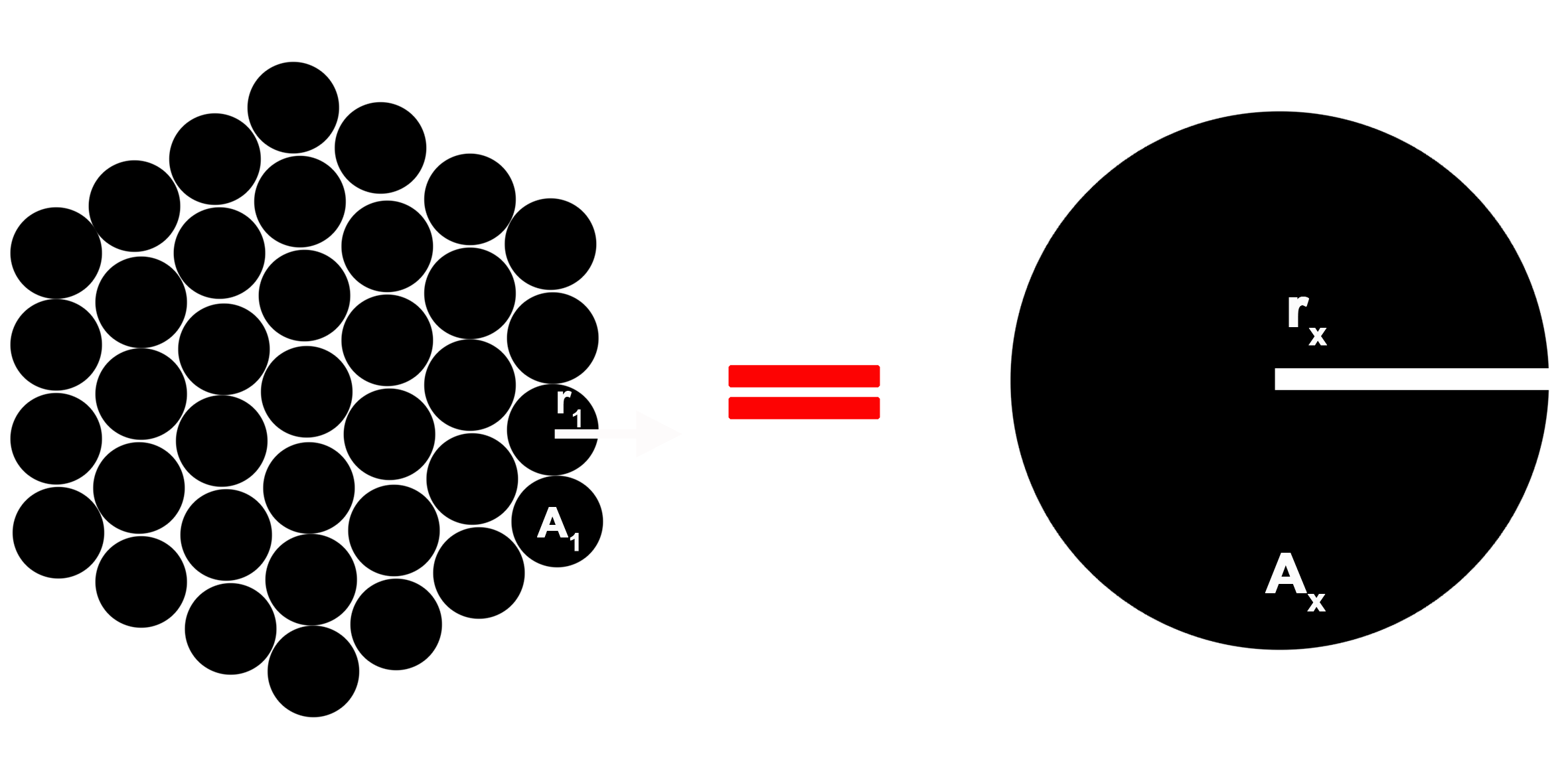

Supplement: Figure S3 — Theoretical diameter and cross-section area of the braided suture. Accurate knowledge of the diameters is important for defining a cross-section area of the suture when calculating the stress in biomechanical tests. Based on single fibre diameters, a theoretical diameter was calculated using the following equation. The cross-section area A of a circle with radius r is defined as, (1); The cross-section area Ax as a sum of several single cross-section areas A1 is defined as, (2); After the insertion after formula 1, (3); Rearrangement yields (4); The calculated radius is used to define an approximated cross-section area for braided sutures of spider silk. Figure S3 illustrates the calculation above. (TIF) [file pone.0061100.s003.tif]
